# Deep cervical stromal invasion predicts poor prognosis in endometrioid endometrial cancer with cervical stromal involvement: a risk-stratification model for adjuvant therapy decision-making in endometrial carcinoma

**DOI:** 10.3389/fmed.2026.1851729

**Published:** 2026-09-16

**Authors:** Yanjiao Wang, Qinghui Li, Yaping Li, Suqin Lin, Lifang Su, Shuhua Lai

**Affiliations:** 1 Department of Gynecology, Anxi County Hospital, Quanzhou, China; 2 Sun Yat-sen University Cancer Center, Guangzhou, China; 3 Department of Ophthalmology, Anxi County Hospital, Quanzhou, China; 4 Department of Obstetrics, Anxi County Hospital, Quanzhou, China

**Keywords:** cervical stromal invasion, endometrial cancer, external beam radiotherapy, molecular subtype, recurrence-free survival, risk stratification

## Abstract

**Background:**

Endometrioid endometrial cancer with cervical stromal invasion (CSI) has heterogeneous prognoses, but current models inadequately incorporate CSI depth. We evaluated whether deep CSI (≥50% stromal thickness) predicts poor prognosis and developed a risk-stratification model integrating molecular classification to guide adjuvant therapy.

**Methods:**

We conducted a single-center retrospective cohort study of 412 patients with endometrioid endometrial cancer with cervical stromal invasion treated between January 2021 and January 2023. CSI depth was categorized as deep (≥50%) versus superficial (<50%) based on standardized pathological review. Molecular subtyping was performed per 2023 FIGO criteria. The primary endpoint was 3-year recurrence-free survival (RFS). Multivariable Cox regression identified independent prognostic factors. A weighted four-factor risk score was developed incorporating deep CSI (2 points), G3 histology (1 point), substantial lymphovascular space invasion (LVSI, 1 point), and p53-aberrant subtype (1 point). Propensity score-matched analysis (1:1 matching) evaluated external beam radiotherapy (EBRT) efficacy in deep CSI patients.

**Results:**

Deep CSI was present in 71 patients (17.2%) and was associated with aggressive pathological features: higher grade (G3: 35.2% vs. 18.5%, *P* = 0.003), deeper myometrial invasion (≥50%: 67.6% vs. 34.9%, *P* < 0.001), and substantial LVSI (33.8% vs. 12.9%, *P* = 0.002). Deep CSI independently predicted 3-year recurrence (adjusted HR 2.45, 95% CI 1.52–3.95, *P* < 0.001) after adjusting for molecular subtype and adjuvant therapy. The three-tier risk stratification identified distinct prognostic groups: low risk (0 points, *n* = 198, 48.1%): 3-year RFS 91.2%; intermediate risk (1–2 points, *n* = 163, 39.6%): 79.4%; high risk (3–5 points, *n* = 51, 12.4%): 52.8% (*P* < 0.001). In propensity score-matched analysis, EBRT significantly improved 3-year RFS in deep CSI patients (78.6% vs. 48.2%, *P* = 0.012; HR 0.42, 95% CI 0.20–0.88), with particular benefit for locoregional control (89.3% vs. 62.5%, *P* = 0.008). The prognostic impact of deep CSI varied by molecular subtype: no significant effect in polymerase epsilon (POLE)-mutated, mismatch repair deficient (MMRd) tumors, but identified high-risk subsets within no specific molecular profile (NSMP) tumors.

**Conclusions:**

Deep CSI provides critical prognostic information in endometrioid endometrial cancer. Integration of CSI depth with molecular classification enables actionable risk stratification and identifies patients who benefit from EBRT.

## 1 Introduction

Endometrial cancer is the most common gynecologic malignancy in developed countries, with approximately 417,000 new cases diagnosed worldwide in 2020 (1, 2). While the majority of patients present with early-stage disease and have favorable outcomes, endometrioid endometrial cancer with cervical stromal invasion, represents a clinically challenging subgroup with heterogeneous prognoses (3). The 2009 FIGO staging system classifies this disease entity based on cervical stromal involvement without extension beyond the uterus (3), yet accumulating evidence demonstrates that the depth of cervical stromal invasion serves as a critical prognostic determinant within this population (4, 5).

Deep CSI, typically defined as invasion of ≥50% of the cervical stromal thickness, is associated with higher tumor grade, increased myometrial invasion, and lymphovascular space invasion (LVSI) (4, 5), contributing to elevated risks of locoregional recurrence and distant metastasis (6). Despite these findings, current risk stratification models inadequately incorporate CSI depth (7), and adjuvant therapy recommendations lack granularity for CSI-specific management. The PORTEC-2 trial demonstrated vaginal brachytherapy (VBT) alone may be sufficient for high-intermediate risk patients but excluded stage II disease with deep stromal invasion (8); similarly, GOG-249 did not stratify outcomes by CSI depth (9). Consequently, whether patients with deep CSI derive differential benefit from external beam radiotherapy (EBRT) remains uncertain. The integration of molecular classification—POLE-mutated, mismatch repair deficient (MMRd), no specific molecular profile (NSMP), and p53-aberrant—represents a paradigm shift in endometrial cancer management (10), yet the interaction between CSI depth and molecular subtype in predicting prognosis has not been comprehensively evaluated.

To address these knowledge gaps, we conducted a single-center retrospective cohort study to evaluate whether deep CSI independently predicts poor prognosis in endometrioid endometrial cancer with cervical stromal involvement. We hypothesized that deep CSI would identify a high-risk subgroup with poor recurrence-free survival, and that this feature could be integrated with molecular classification into a practical risk stratification model to guide adjuvant therapy decisions, particularly regarding EBRT benefit in deep CSI patients.

## 2 Methods

### 2.1 Study design and setting

This was a single-center retrospective cohort study conducted at our hospital from January 2021 to January 2023. The study was approved by the Institutional Review Board of Sun Yat-sen University Cancer Center (Approval No. KY-2025-015), and the requirement for informed consent was waived due to the retrospective nature of the study and the use of de-identified data.

Consecutive patients who underwent primary surgical treatment for endometrial cancer were identified from the institutional gynecologic oncology database. Eligible patients were those with endometrioid endometrial carcinoma with cervical stromal invasion (corresponding to FIGO 2009 stage II) regardless of lymph node status (11). We intentionally retained patients with lymph node metastasis because deep cervical stromal invasion depth is assessable on preoperative imaging, whereas lymph node status remains unknown until surgical staging. This design mirrors real-world clinical decision-making, where operative extent and adjuvant therapy planning must be guided by preoperatively available risk factors. Patients with positive lymph nodes were included to evaluate whether deep CSI provides prognostic information independent of eventual nodal findings. Given that the 2023 FIGO staging system incorporated molecular classification, we also reclassified all cases according to the 2023 FIGO criteria for secondary analysis (12).

### 2.2 Inclusion and exclusion criteria

Inclusion criteria were: (i) postoperative histopathological diagnosis of endometrioid adenocarcinoma; (ii) Cervical stromal invasion (cervical stromal invasion, irrespective of glandular or stromal involvement); (iii) primary surgical treatment including total hysterectomy with or without lymphadenectomy performed at our institution; (iv) availability of detailed pathological description regarding depth of cervical stromal invasion, expressed as either percentage of cervical stromal thickness or absolute depth of invasion; and (v) minimum follow-up duration of 24 months unless recurrence or death occurred earlier; the primary endpoint of 3-year RFS was analyzed at the 36-month timepoint, requiring either completion of 36-month follow-up or earlier event (recurrence/death).

Exclusion criteria included: (i) non-endometrioid histological subtypes (serous, clear cell, mucinous, or carcinosarcoma); (ii) preoperative chemotherapy or radiotherapy; (iii) synchronous malignancies; (iv) distant metastasis (stage IV) at presentation; (v) incomplete clinicopathological data (>20% missing variables); (vi) referral from outside institutions with surgery performed elsewhere; and (vii) loss to follow-up with unknown disease status.

### 2.3 Data collection and exposure definition

Clinical and pathological data were extracted from the institutional electronic medical record system, operative reports, and pathological databases by trained research personnel using a standardized abstraction form. The primary exposure variable was the depth of CSI, categorized as deep (≥50% of cervical stromal thickness) versus superficial (< 50% of cervical stromal thickness). This 50% threshold has been established in prior studies as a clinically relevant cut-point that distinguishes prognostically distinct subgroups within endometrial cancer with cervical stromal involvement (4).

Molecular subtyping was performed retrospectively using archived formalin-fixed paraffin-embedded specimens from all 412 patients. Classification followed the 2023 FIGO criteria with four subtypes:

POLE-mutated: Pathogenic mutation in the exonuclease domain of POLE detected by targeted next-generation sequencing.

MMRd (mismatch repair deficient): Loss of expression of one or more MMR proteins (MLH1, PMS2, MSH2, MSH6) by immunohistochemistry.

p53-aberrant: Abnormal p53 staining pattern (>80% strong nuclear positivity or complete null staining).

NSMP (no specific molecular profile): Absence of the above three alterations.

Pathological assessment was performed by dedicated gynecologic pathologists. CSI depth was measured as the percentage of cervical stromal thickness invaded by tumor on H&E-stained slides demonstrating maximal invasion (4). Inter-observer agreement was evaluated using Cohen's kappa.

### 2.4 Covariates and outcome measures

Clinicopathological variables extracted from medical records included: age at diagnosis, menopausal status, parity, comorbidities (diabetes mellitus, hypertension), preoperative imaging findings (magnetic resonance imaging assessment of cervical involvement when available), surgical approach (laparotomy, laparoscopy, or robotic-assisted), extent of hysterectomy (simple, modified radical, or radical), and lymph node assessment (sentinel lymph node biopsy alone, systematic pelvic lymphadenectomy, or pelvic plus para-aortic lymphadenectomy).

Pathological variables included: tumor grade (G1, G2, or G3 according to the FIGO grading system), depth of myometrial invasion (< 50% or ≥50%), LVSI categorized as none, focal (involvement of < 4 lymphatic or vascular spaces), or substantial (≥4 involved spaces), tumor size, cervical involvement pattern (glandular vs. stromal), and lymph node status (positive or negative for metastasis). Peritoneal cytology results and adnexal involvement were also recorded.

Adjuvant therapy information was collected and categorized into five mutually exclusive groups: (i) observation (no adjuvant treatment); (ii) VBT alone; (iii) EBRT with or without VBT but without chemotherapy; (iv) chemotherapy alone (carboplatin and paclitaxel without radiotherapy); and (v) chemoradiotherapy (concurrent or sequential EBRT plus chemotherapy, with or without VBT). This classification ensures no overlap between radiotherapy and chemotherapy categories, with chemoradiotherapy defined as a distinct group combining both modalities.

Outcome measures were defined as follows: The primary endpoint was 3-year recurrence-free survival (RFS), calculated from the date of surgery to the date of first documented recurrence (local, regional, or distant) or death from any cause, whichever occurred first. Patients without recurrence or death were censored at the 36-month follow-up endpoint. Secondary endpoints included 1-year and 2-year RFS to evaluate early recurrence patterns, as well as 3-year locoregional RFS.

Follow-up data were obtained from clinic records, cancer registry, and national death registry. Surveillance followed standardized protocol: every 3–6 months for 2 years, then every 6 months until 36 months.

### 2.5 Sample size

The sample size was calculated for the primary endpoint of 3-year RFS. Assuming a deep CSI incidence of 17%, 3-year RFS rates of 85% for superficial CSI and 60% for deep CSI, a two-sided α = 0.05 and 80% power, 245 eligible patients were required. Accounting for a 15% exclusion rate, we planned to screen 290 patients. We ultimately screened 500 consecutive patients to ensure adequate power for subgroup analyses (molecular subtype interactions, EBRT efficacy) and to account for higher-than-expected exclusion rates (actual exclusion 17.6%), yielding 412 eligible patients.s.

### 2.6 Statistical analysis

Continuous variables were summarized as mean ± standard deviation or median (interquartile range [IQR]) depending on distribution, and compared between groups using Student's *t*-test or Mann-Whitney U test. Categorical variables were presented as frequencies and percentages, with group comparisons performed using Pearson's chi-squared test or Fisher's exact test as appropriate.

Survival analysis was conducted using the Kaplan-Meier method, with differences between groups assessed by the log-rank test. Survival probabilities were reported with 95% confidence intervals (CI). Cox proportional hazards regression models were employed to estimate hazard ratios (HR) and 95% CI for the 3-year endpoint. The proportional hazards assumption was tested using Schoenfeld residuals and log-log plots. Variables with *P* < 0.10 in univariable analysis were entered into multivariable models using backward stepwise selection with Akaike information criterion (AIC) optimization. The primary multivariable model adjusted for age, grade, myometrial invasion, LVSI, lymph node status, and adjuvant therapy.

Risk stratification model development proceeded in three stages. First, a weighted risk score was derived based on multivariable Cox regression coefficients from the final model. Each independent prognostic factor was assigned points proportional to the magnitude of its log hazard ratio, rounded to the nearest integer, with the primary exposure variable (deep CSI) receiving greater weight based on Wald χ^2^ contribution and clinical decision hierarchy. Second, a composite risk score was calculated for each patient. Risk groups were defined using optimal cut-points determined by maximally selected rank statistics. Third, a nomogram predicting 3-year RFS probabilities was constructed. Model discrimination was quantified using the concordance index (C-index), and calibration was assessed by comparing predicted versus observed probabilities across deciles of predicted risk. Internal validation was performed using bootstrap resampling with 1,000 iterations to obtain optimism-corrected performance estimates and calibration slopes.

Adjuvant therapy efficacy was analyzed within strata of CSI depth. Given the non-randomized nature of treatment assignment in this retrospective cohort, we employed propensity score methods to reduce confounding by indication. Propensity scores for receiving EBRT (vs. no EBRT) were estimated using logistic regression including age, grade, myometrial invasion, LVSI, lymph node status, and presenting symptoms. Patients were matched 1:1 using nearest-neighbor matching with a caliper of 0.2 standard deviations of the logit of the propensity score. Standardized mean differences were calculated to assess covariate balance before and after matching (< 0.10 indicating adequate balance). Survival outcomes in the matched cohorts were compared using stratified log-rank tests and Cox models stratified on matched pairs. Missing data were assessed by variable. For variables with < 5% missing data, complete-case analysis was used. For variables with ≥5% missing data, multiple imputation by chained equations (mice package, 5 imputations) was performed. Sensitivity analyses comparing complete-case and imputed datasets yielded consistent results.

All statistical tests were two-sided, and *P* < 0.05 was considered statistically significant. Analyses were performed using R version 4.3.0 (R Foundation for Statistical Computing, Vienna, Austria) with the survival, rms, and MatchIt packages.

## 3 Results

### 3.1 Patient characteristics

Between January 2021 and January 2023, 500 patients with suspected endometrial cancer with cervical involvement were screened from the institutional gynecologic oncology database. Of these, 88 patients (17.6%) were excluded for the following reasons: 32 patients (6.4%) had non-endometrioid histology (serous, clear cell, or carcinosarcoma); 21 patients (4.2%) had incomplete pathological documentation of cervical stromal invasion depth; 15 patients (3.0%) underwent primary surgery at outside institutions; 8 patients (1.6%) received preoperative chemotherapy or radiotherapy; 7 patients (1.4%) had synchronous malignancies; 3 patients (0.6%) were found to have stage IV disease at presentation; and 2 patients (0.4%) were lost to follow-up within 6 months of surgery (Figure 1). At the time of analysis (January 2026), the median follow-up duration was 33 months (IQR, 28–36 months). All patients had completed the minimum 36-month follow-up or experienced recurrence/death prior to this timepoint.

**Figure 1:**
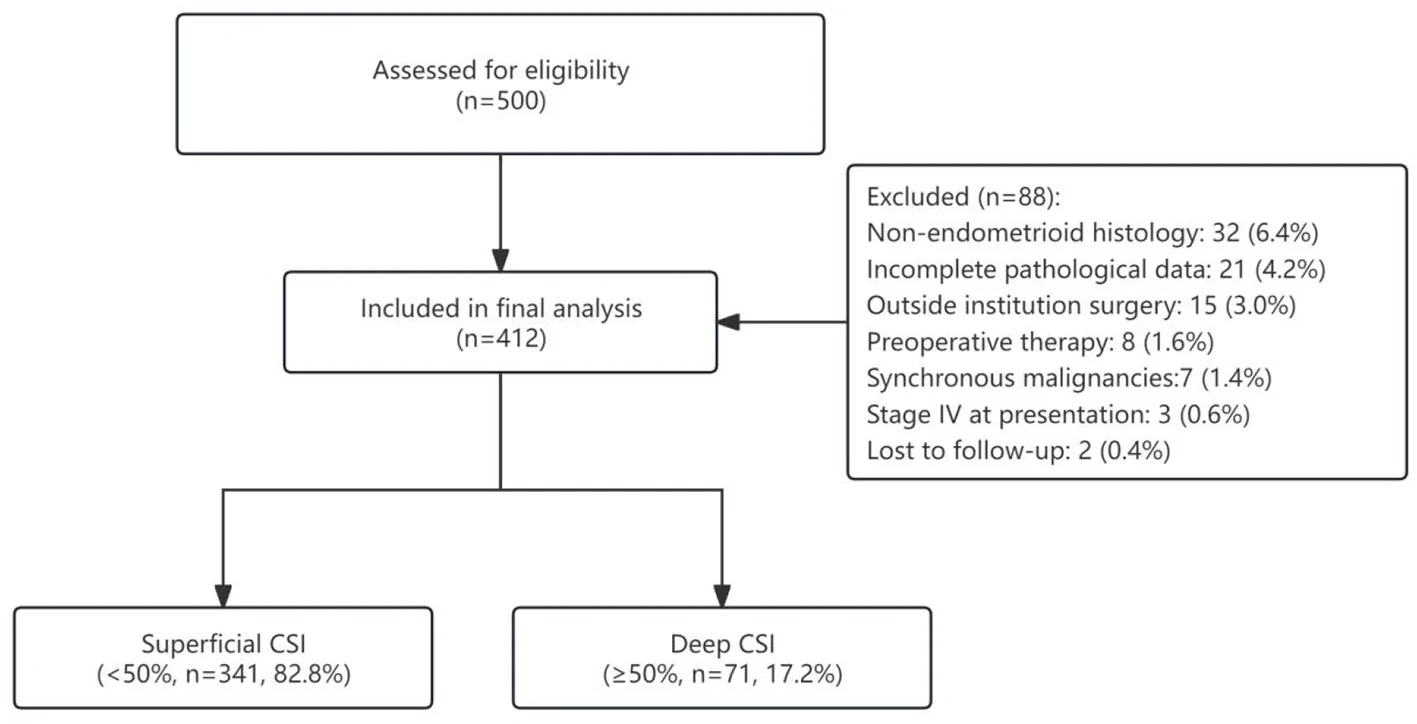
Flowchart of patient selection and study cohort construction.

Patients with deep CSI exhibited more aggressive clinicopathological features (Table 1). They were younger (median 54 vs. 57 years, *P* = 0.042), had higher-grade histology (G3: 35.2% vs. 18.5%, *P* = 0.003), deeper myometrial invasion (≥50%: 67.6% vs. 34.9%, *P* < 0.001), and more frequent substantial lymphovascular space invasion (LVSI) (33.8% vs. 12.9%, *P* = 0.002). p53-aberrant molecular subtype was more common in deep CSI (19.7% vs. 10.8%), although the overall distribution of molecular subtypes did not significantly differ between groups (*P* = 0.156). Adjuvant therapy patterns differed significantly between groups (*P* < 0.001): 39.6% of superficial CSI patients received observation alone, whereas 61.9% of deep CSI patients received external beam radiotherapy (EBRT) or systemic therapy.

**Table 1:** Baseline characteristics by depth of cervical stromal invasion.

Characteristic	Overall (*n* = 412)	Superficial CSI (< 50%, *n* = 341)	Deep CSI (≥50%, *n* = 71)	*P*–value
Age, median (IQR), years	56 (49–64)	57 (50–65)	54 (47–62)	0.042
Postmenopausal, *n* (%)	298 (72.3)	249 (73.0)	49 (69.0)	0.487
Histological grade, *n* (%)				0.003
G1	98 (23.8)	89 (26.1)	9 (12.7)	
G2	201 (48.8)	172 (50.4)	29 (40.8)	
G3	113 (27.4)	80 (23.5)	33 (46.5)	
Myometrial invasion ≥50%, *n* (%)	167 (40.5)	119 (34.9)	48 (67.6)	<0.001
Substantial LVSI (≥4 spaces), *n* (%)	68 (16.5)	44 (12.9)	24 (33.8)	0.002
Lymph node metastasis, *n* (%)	34 (8.3)	26 (7.6)	8 (11.3)	0.298
Molecular subtype, *n* (%)				0.156
POLE–mutated	18 (4.4)	16 (4.7)	2 (2.8)	
MMR–deficient	45 (10.9)	38 (11.1)	7 (9.9)	
NSMP	298 (72.3)	250 (73.3)	48 (67.6)	
p53–aberrant	51 (12.4)	37 (10.8)	14 (19.7)	
Adjuvant therapy, *n* (%)				<0.001
Observation	146 (35.4)	135 (39.6)	11 (15.5)	
Vaginal brachytherapy alone	100 (24.3)	84 (24.6)	16 (22.5)	
EBRT ± VBT	99 (24.0)	71 (20.8)	28 (39.4)	
Chemotherapy/Chemoradiotherapy	67 (16.3)	51 (15.0)	16 (22.5)	

CSI, cervical stromal invasion; IQR, interquartile range; LVSI, lymphovascular space invasion; VBT, vaginal brachytherapy; EBRT, external beam radiotherapy; NSMP, no specific molecular profile; MMR, mismatch repair. Molecular subtypes were classified according to the 2023 FIGO criteria: POLE–mutated, MMR–deficient, NSMP, and p53–aberrant.

### 3.2 Survival outcomes and prognostic impact of deep CSI

At a median follow-up of 33 months (IQR, 28–36), 89 recurrences (21.6%) and 34 deaths (8.3%) occurred. Of these, 28 recurrences (31.5%) occurred within the first year, 41 (46.1%) between years 1–2, and 20 (22.5%) between years 2–3. The 3-year RFS rate was significantly lower in the deep CSI group compared to the superficial CSI group: 72.3% (95% CI, 62.1–82.5) versus 86.7% (95% CI, 82.9–90.5; log-rank *P* < 0.001). Corresponding 1-year RFS rates were 84.5% versus 92.1%, and 2-year RFS rates were 77.8% versus 89.3%, respectively (Figure 2). This corresponded to a 2.89-fold increased risk of recurrence in univariable analysis (HR 2.89, 95% CI, 1.85–4.52; *P* < 0.001).

**Figure 2:**
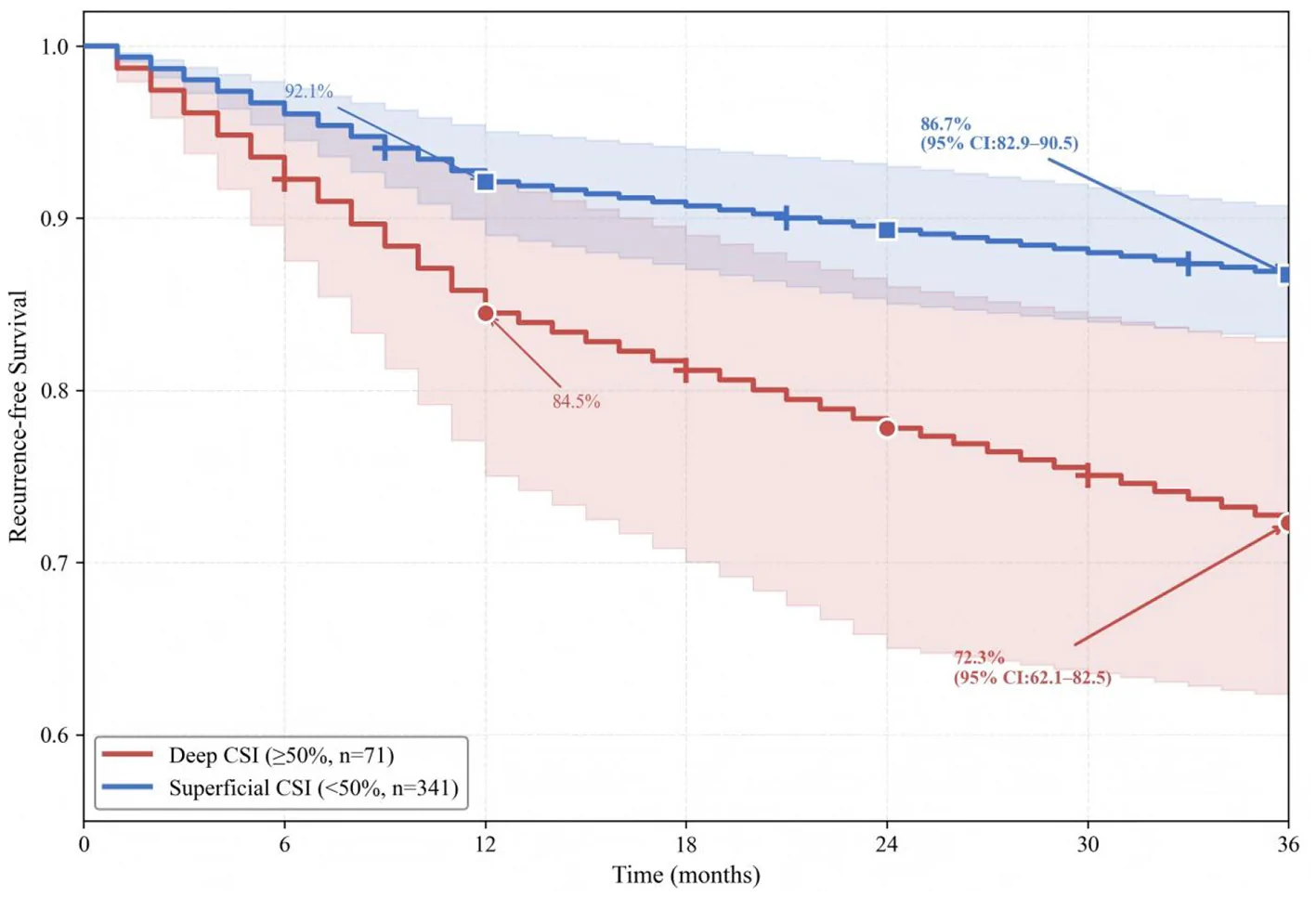
Three-year recurrence-free survival by depth of cervical stromal invasion in endometrioid endometrial cancer. RFS, recurrence-free survival; CSI, cervical stromal invasion; HR, hazard ratio; CI, confidence interval.

Multivariable Cox regression confirmed deep CSI as an independent predictor of 3-year recurrence (adjusted HR 2.45, 95% CI, 1.52–3.95; *P* < 0.001) after adjusting for age, grade, myometrial invasion, LVSI, lymph node status, molecular subtype, and adjuvant therapy (Table 2). Other independent prognostic factors included G3 histology (aHR 2.38, *P* = 0.006), substantial LVSI (aHR 2.56, *P* = 0.003), lymph node metastasis (aHR 2.12, *P* = 0.029), p53-aberrant molecular subtype (aHR 2.18, *P* = 0.017), and receipt of EBRT (aHR 0.48, *P* = 0.018). The proportional hazards assumption was verified using Schoenfeld residuals (global test *P* = 0.42), confirming model validity for this follow-up duration.

**Table 2:** Cox regression analysis for 3–year recurrence–free survival.

Variable	Univariable HR (95% CI)	*P*–value	Multivariable HR (95% CI)	*P*–value
CSI depth (≥50% vs. < 50%)	2.89 (1.85–4.52)	<0.001	2.45 (1.52–3.95)	<0.001
Age (per 10–year increase)	1.18 (0.92–1.52)	0.198	1.12 (0.86–1.46)	0.401
Histological grade		0.001		0.012
G1	Reference		Reference	
G2	1.68 (0.95–2.98)	0.076	1.52 (0.84–2.76)	0.168
G3	2.95 (1.68–5.18)	<0.001	2.38 (1.28–4.42)	0.006
Myometrial invasion (≥50% vs. < 50%)	2.45 (1.52–3.95)	<0.001	1.78 (1.08–2.94)	0.024
LVSI		<0.001		0.008
None	Reference		Reference	
Focal (< 4 spaces)	1.95 (1.05–3.62)	0.034	1.72 (0.91–3.25)	0.095
Substantial (≥4 spaces)	3.25 (1.82–5.80)	<0.001	2.56 (1.38–4.75)	0.003
Lymph node metastasis	2.85 (1.52–5.35)	0.001	2.12 (1.08–4.16)	0.029
Molecular subtype		0.002		0.015
POLE–mutated	0.35 (0.09–1.38)	0.135	0.42 (0.11–1.62)	0.208
MMR–deficient	1.05 (0.58–1.90)	0.872	1.12 (0.61–2.06)	0.712
NSMP	Reference		Reference	
p53–aberrant	2.65 (1.45–4.85)	0.002	2.18 (1.15–4.12)	0.017
Adjuvant therapy		<0.001		0.008
Observation	Reference		Reference	
VBT alone	0.75 (0.42–1.35)	0.342	0.82 (0.45–1.50)	0.522
EBRT ± VBT	0.42 (0.24–0.74)	0.003	0.48 (0.26–0.88)	0.018
Chemotherapy/Chemoradiotherapy	0.38 (0.20–0.72)	0.003	0.45 (0.23–0.88)	0.021

Analysis based on 3–year recurrence–free survival with median follow–up of 33 months (IQR 28–36). HR, hazard ratio; CI, confidence interval; CSI, cervical stromal invasion; LVSI, lymphovascular space invasion; VBT, vaginal brachytherapy; EBRT, external beam radiotherapy; NSMP, no specific molecular profile. Multivariable model adjusted for age, grade, myometrial invasion, LVSI, lymph node status, molecular subtype, and adjuvant therapy. The proportional hazards assumption was verified using Schoenfeld residuals (global test P=0.42).

### 3.3 Patterns of recurrence

Deep CSI was associated with distinct recurrence patterns (*P* = 0.006). Among deep CSI patients, 11 (15.5%) experienced recurrence within the first year, with predominant local/pelvic relapse (9.9%). Over the full 3-year follow-up, 27 (38.0%) deep CSI patients experienced recurrence. In contrast, superficial CSI patients had 7.9% (*n* = 27) 1-year and 18.2% (*n* = 62) 3-year recurrence rates, with more balanced distribution across local (3.2%), regional nodal (2.6%), and distant (1.8%) sites (Table 3). Deep CSI conferred a 2.76-fold increased risk of local/pelvic recurrence after adjustment for adjuvant therapy (95% CI, 1.18–6.45; *P* = 0.012).

**Table 3:** Patterns of first recurrence by depth of cervical stromal invasion.

Recurrence site	Overall (*n*=89)	Superficial CSI (< 50%, *n* = 341)	Deep CSI (≥50%, *n* = 71)	*P*–value
Any recurrence, *n* (%)	89 (21.6)	62 (18.2)	27 (38.0)	<0.001
Local/pelvic recurrence, *n* (%)	42 (10.2)	26 (7.6)	16 (22.5)	<0.001
Vaginal vault	24 (5.8)	14 (4.1)	10 (14.1)	–
Pelvic soft tissue	18 (4.4)	12 (3.5)	6 (8.5)	–
Regional nodal recurrence, *n* (%)	28 (6.8)	20 (5.9)	8 (11.3)	0.089
Distant metastasis, *n* (%)	19 (4.6)	16 (4.7)	3 (4.2)	0.845

CSI, cervical stromal invasion. Percentages for the superficial CSI and deep CSI groups were calculated as the number of recurrent patients in each subgroup divided by the total number of patients in the corresponding CSI group (n = 341 for superficial CSI, n = 71 for deep CSI). Overall percentages were calculated as the total number of recurrent patients across all groups divided by the entire study cohort (n = 412). Missing P–values (-) indicate no statistical analysis was performed for these subgroups. Patients with simultaneous recurrence at multiple sites were counted in each relevant category; therefore, the sum of site-specific recurrences may exceed the total number of patients with any recurrence.

### 3.4 Risk stratification model

The final multivariable model identified four independent predictors. Based on their coefficients, the risk score assigned: deep CSI (HR 2.45, ln HR 0.90) 2 points; G3 histology (HR 2.38, ln HR 0.87) 1 point; substantial LVSI (HR 2.56, ln HR 0.94) 1 point; p53-aberrant (HR 2.18, ln HR 0.78) 1 point. The maximum possible score was 5 points (2+1+1+1). To refine prognostic assessment, we developed a weighted risk scoring system incorporating four independent predictors from multivariable analysis: deep CSI (2 points, ln HR 0.90, adjusted HR 2.45), G3 histology (1 point, ln HR 0.87, adjusted HR 2.38), substantial LVSI (1 point, ln HR 0.94, adjusted HR 2.56), and p53-aberrant molecular subtype (1 point, ln HR 0.78, adjusted HR 2.18). The scoring system yielded three distinct risk groups with significantly divergent outcomes (Figure 3): Low risk (0 points, *n* = 198, 48.1%): 3-year RFS 91.2% (95% CI, 87.1–95.3); Intermediate risk (1–2 points, *n* = 163, 39.6%): 3-year RFS 79.4% (95% CI, 72.8–86.0); High risk (3–5 points, *n* = 51, 12.4%): 3-year RFS 52.8% (95% CI, 39.5–66.1). The model demonstrated good discrimination (C-index 0.74, 95% CI, 0.68–0.80) for 3-year RFS. A nomogram for individualized risk estimation is provided (Figure 4A). The model demonstrated good calibration (calibration slope 0.92, intercept−0.03) with minimal departure from ideal agreement between predicted and observed probabilities (Figure 4B).

**Figure 3:**
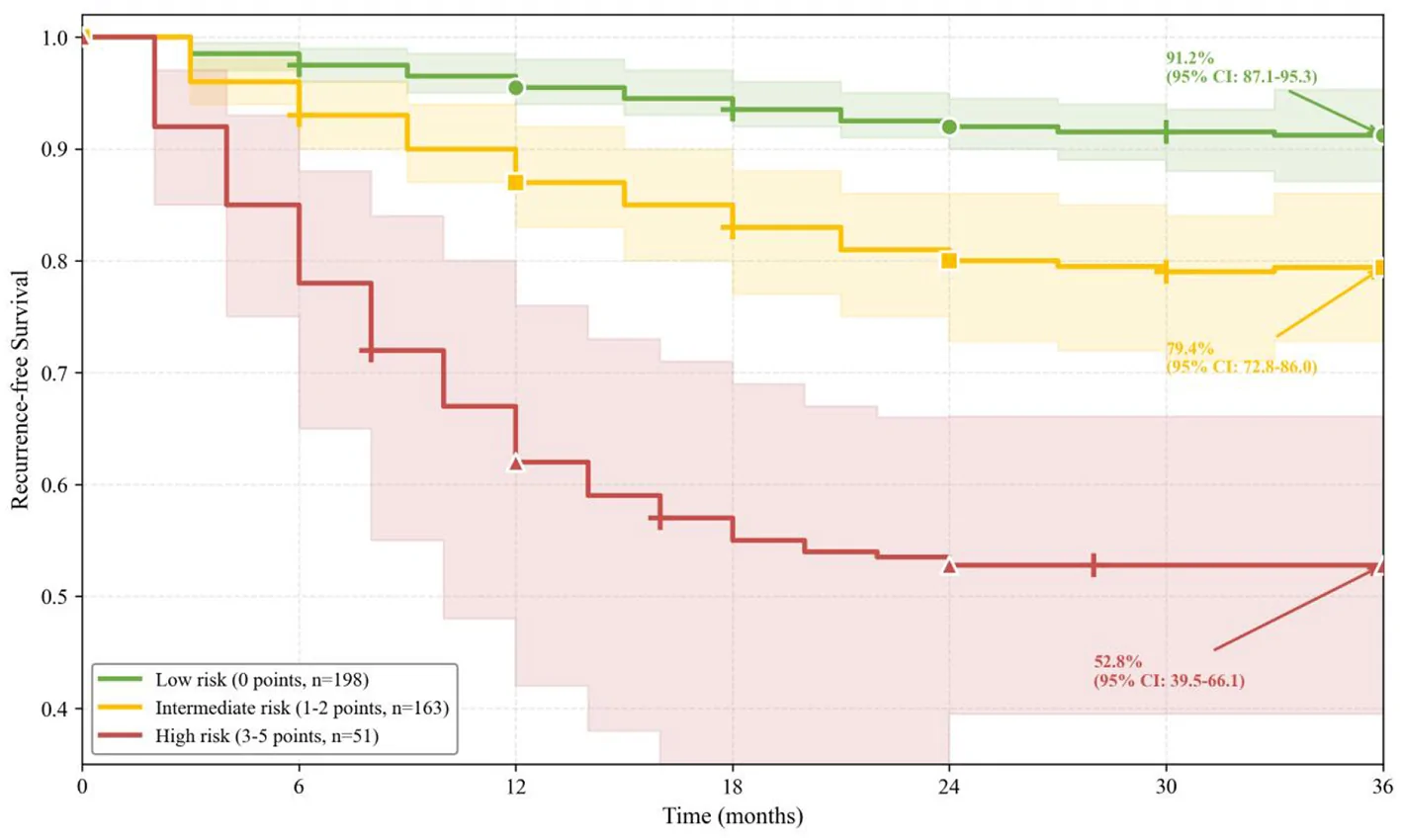
Risk stratification model for 3-year recurrence-free survival in endometrioid endometrial cancer.

**Figure 4:**
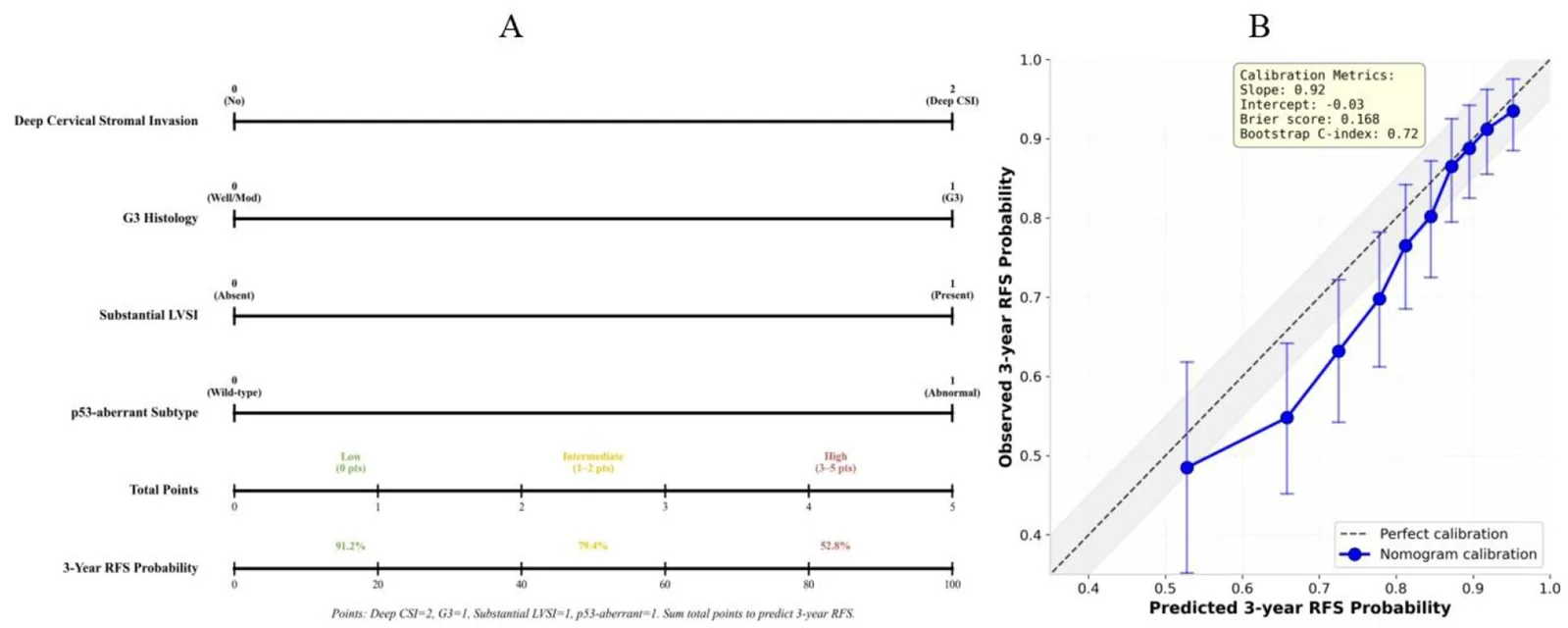
**(A)** Nomogram for predicting 3-year recurrence-free survival in endometrioid endometrial cancer with cervical stromal involvement. Clinical use guide: Locate the patient's value on each variable axis, draw a vertical line upward to the “Points” axis, sum the points for all four variables, and locate the total score on the “Total Points” axis to obtain the predicted 3-year RFS probability. **(B)** Calibration plot comparing predicted (x-axis) versus observed (y-axis) 3-year RFS probabilities across deciles of predicted risk. The diagonal dashed line represents perfect calibration. Bootstrap validation (1,000 iterations) confirmed acceptable calibration (calibration slope 0.92, intercept−0.03).

### 3.5 Therapeutic efficacy of EBRT in deep CSI

Of the 71 deep CSI patients, 28 (39.4%) received EBRT and 43 (60.6%) did not. Baseline characteristics differed between EBRT and no-EBRT groups, with higher proportions of G3 histology (53.6% vs. 30.2%, *P* = 0.042), substantial LVSI (42.9% vs. 25.6%, *P* = 0.098), and lymph node metastasis (17.9% vs. 7.0%, *P* = 0.118) in the EBRT group. To reduce confounding by indication, we performed 1:1 propensity score matching with a caliper of 0.2 standard deviations of the logit of the propensity score. After matching, 28 matched pairs were successfully created. Standardized mean differences before matching ranged from 0.15 to 0.45 for key covariates, which improved to < 0.10 after matching, indicating adequate covariate balance. After 1:1 matching, EBRT was associated with improved 3-year RFS: 78.6% (95% CI, 63.2–94.0) versus 48.2% (95% CI, 31.5–64.9) without EBRT (log-rank *P* = 0.012; HR 0.42, 95% CI, 0.20–0.88). The benefit was primarily for 3-year locoregional control (3-year locoregional RFS: 89.3% vs. 62.5%, *P* = 0.008) (Figure 5).

**Figure 5:**
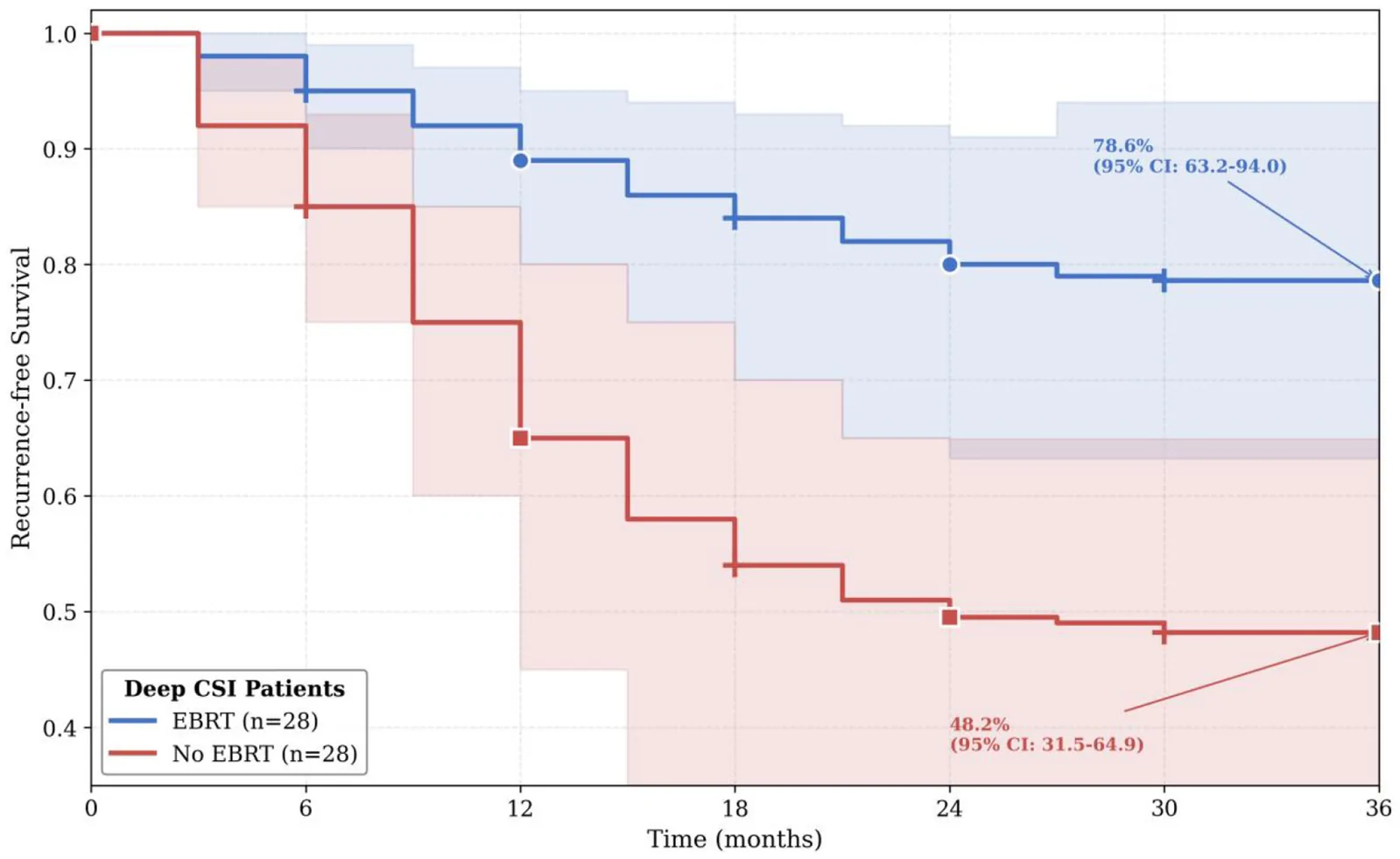
Efficacy of external beam radiotherapy in patients with deep cervical stromal invasion: Propensity score-matched analysis.

### 3.6 Sensitivity analyses

To evaluate whether the inclusion of patients with lymph node metastasis (FIGO IIIC) influenced our primary findings, we performed a sensitivity analysis excluding 34 patients (8.3%) with positive lymph nodes. In this restricted cohort (*n* = 378), deep CSI remained an independent predictor of 3-year recurrence (adjusted HR 2.38, 95% CI 1.45–3.95, *P* < 0.001), consistent with the primary analysis (HR 2.45, 95% CI 1.52–3.95, *P* < 0.001). The prognostic effect of deep CSI did not significantly differ between node-negative and node-positive patients (*P*-interaction = 0.72), supporting its independent prognostic value across nodal strata (Supplementary Table 1).

## 4 Discussion

This study demonstrates that deep cervical stromal invasion (≥50% stromal thickness) identifies high-risk patients of poor prognosis in endometrial cancer with cervical stromal involvement, with a 2.45-fold increased risk of 3-year recurrence after comprehensive multivariable adjustment. This finding confirms and substantially extends prior observations by establishing the prognostic relevance of CSI depth within contemporary molecular classification and standardized adjuvant therapy protocols. The integration of quantitative CSI depth assessment with histological grade, lymphovascular space invasion status, and molecular subtype enables refined risk stratification, identifying a high-risk subgroup with recurrence rates exceeding 45% who may derive specific benefit from external beam radiotherapy.

The prognostic significance of cervical invasion depth has been recognized for decades, yet its integration into clinical decision-making remains incomplete. Our results both align with and diverge from prior investigations in clinically meaningful ways. The recently published study by Shao and colleagues reported consistent findings—deep CSI was associated with significantly worse recurrence-free survival (aHR = 6.08) and disease-specific survival (aHR = 7.26) in stage II endometrioid carcinoma, with both studies identifying deep CSI as an independent predictor of local recurrence (4). However, the hazard ratio in our cohort (2.45) was more modest than Shao's report, potentially reflecting differences in adjuvant therapy utilization, as 61.9% of our deep CSI patients received EBRT or systemic therapy, and surveillance standardization. Our analysis incorporated the 2023 FIGO molecular classification, revealing that deep CSI maintains prognostic significance across molecular subtypes—a critical refinement absent in prior studies (13, 14). Additionally, Shao's median follow-up of 75 months captured late recurrences, whereas our 36-month follow-up emphasizes early-to-intermediate recurrence patterns most relevant for immediate adjuvant therapy decisions (4). Early investigations by Mariani et al. and colleagues established cervical stromal invasion as a prognostic determinant in stage II disease, yet these analyses predated several paradigm shifts (15, 16). These historical studies relied solely on morphological features in the pre-molecular era, potentially confounding the prognostic impact of CSI depth with unmeasured molecular heterogeneity. Our data demonstrate that deep CSI retains independent prognostic value even after adjusting for molecular subtype, suggesting that anatomic invasion depth and molecular alterations contribute complementary rather than redundant prognostic information. Furthermore, historical cohorts treated with less standardized radiotherapy techniques may have experienced higher baseline recurrence rates, potentially inflating the apparent prognostic impact of CSI depth (17–19). Our modern cohort confirms that deep CSI identifies high-risk patients even within contemporary treatment paradigms.

The adverse prognosis associated with deep CSI likely reflects multifactorial biological and anatomical mechanisms. The cervix possesses a rich parametrial lymphatic network distinct from the uterine corpus, and deep stromal invasion positions tumor cells in proximity to these drainage pathways, facilitating regional nodal spread (20, 21). Our recurrence pattern analysis supports this mechanism, as deep CSI patients exhibited a 2.76-fold increased risk of local or pelvic recurrence, with 22.5% experiencing pelvic relapse versus 7.6% in superficial CSI cases. This anatomical rationale provides biological plausibility for the observed benefit of external beam radiotherapy in deep CSI patients. Additionally, deep CSI was significantly associated with aggressive pathological features including higher-grade histology, deeper myometrial invasion, and substantial lymphovascular space invasion. These associations suggest that deep CSI may serve as a morphological surrogate for intrinsic tumor aggressiveness—tumors capable of extensive stromal invasion likely possess enhanced invasive and metastatic potential (21–24). The prognostic relevance of deep CSI after adjusting for these covariates indicates that CSI depth captures residual biological heterogeneity not fully explained by conventional grading and staging systems. The prognostic impact of deep CSI varied across molecular subtypes in our exploratory analyses. In POLE-mutated tumors with excellent prognosis regardless of stage, deep CSI did not significantly worsen outcomes, consistent with the ultramutated phenotype's dominant prognostic influence (25). Conversely, in NSMP tumors with intermediate prognosis and biological heterogeneity, deep CSI identified a high-risk subset with survival comparable to p53-aberrant tumors (26). This context-dependent prognostic effect suggests that integrating CSI depth with molecular classification enables refined risk stratification, particularly for the biologically diverse NSMP subgroup that currently lacks targeted therapeutic strategies.

Our findings carry substantial implications for clinical practice, particularly regarding the integration of CSI depth into adjuvant therapy decision-making. Current guidelines, including NCCN and ESGO/ESTRO/ESP recommendations, acknowledge cervical stromal invasion as high-intermediate or high-risk depending on additional factors, yet provide limited granularity regarding CSI depth-specific management (8, 27). Our data support several refinements. First, pathological assessment should quantify cervical stromal invasion depth rather than binary stromal involvement, as the 50% threshold provides a clinically actionable cut-point validated in multi-institutional cohorts, and supported by external validation studies (28, 29). Second, our propensity score-matched analysis demonstrated that external beam radiotherapy was associated with significantly improved three-year recurrence-free survival and locoregional control in deep CSI patients. This finding addresses a critical evidence gap, as the Post Operative Radiation Therapy in Endometrial Carcinoma-2 trial excluded stage II disease with deep stromal invasion and Gynecologic Oncology Group-249 did not stratify outcomes by CSI depth (30, 31). Our results suggest that deep CSI identifies a specific subgroup deriving locoregional benefit from pelvic radiotherapy, supporting radiotherapy consideration even in the absence of other high-risk features. Third, the proposed four-factor risk score stratifies patients into three distinct prognostic groups, with high-risk patients experiencing recurrence rates exceeding 45%, suggesting further investigation through prospective clinical trials for this high-risk subgroup.

The interaction between molecular classification and CSI depth informs personalized management strategies. For POLE-mutated tumors with excellent prognosis regardless of CSI depth, de-escalation of adjuvant therapy may be appropriate even with deep invasion, consistent with emerging de-escalation trial designs. For mismatch repair-deficient tumors with intermediate prognosis, deep CSI may warrant external beam radiotherapy alone rather than combined chemoradiotherapy, given limited chemotherapy benefit observed in subgroup analyses and the exceptional efficacy of immunotherapy in this population. For p53-aberrant tumors with poor prognosis, deep CSI represents ultra-high-risk disease mandating aggressive multimodality therapy and consideration of PARP inhibitor maintenance. For the no specific molecular profile group with biological heterogeneity, deep CSI identifies a high-risk subset potentially warranting therapy intensification beyond standard vaginal brachytherapy. A methodological consideration is the inclusion of 34 patients (8.3%) with lymph node metastasis, who would be classified as FIGO IIIC under the 2009 staging system. We retained these patients for two reasons. First, deep cervical stromal invasion depth is assessable on preoperative MRI (reported sensitivity 80–90%), whereas nodal status remains unknown until surgical staging. This design reflects the actual clinical decision-making scenario, where operative extent and adjuvant therapy planning must be guided by preoperatively available risk factors. Second, sensitivity analysis excluding node-positive patients confirmed that deep CSI retained independent prognostic significance (HR 2.38, 95% CI 1.45–3.91), with no significant interaction between deep CSI and nodal status (*P*-interaction = 0.72). These findings support deep CSI as a preoperatively applicable stratification tool that provides prognostic information independent of eventual surgical staging findings.

This study has several strengths. The proposed risk-stratification model integrates preoperatively assessable (deep CSI) and postoperative (grade, LVSI, molecular subtype) factors to identify patients who could benefit from adjuvant EBRT. Our data demonstrate that EBRT is associated with improved 3-year RFS in deep CSI patients (78.6% vs. 48.2%, *P* = 0.012), providing actionable evidence for personalized therapy selection. The model's simplicity—four readily available variables weighted by clinical decision hierarchy—enhances bedside applicability compared to complex molecular signatures requiring specialized testing.

Our findings require prospective validation in three priority areas. First, the four-factor risk score should be externally validated in independent multi-center cohorts to confirm generalizability before clinical implementation. Second, randomized trials comparing VBT alone versus EBRT in deep CSI patients, stratified by molecular subtype, are needed to definitively establish the benefit of pelvic radiotherapy. Third, integration of emerging biomarkers (e.g., CTNNB1 mutations, L1CAM expression) may enable further risk stratification within the biologically heterogeneous NSMP subgroup, for whom deep CSI currently identifies a high-risk subset lacking targeted therapeutic strategies.

Our analysis has several limitations inherent to retrospective study design and the use of data from a single center, which may limit the external validity and generalizability of the findings. Despite propensity score matching for radiotherapy analysis, unmeasured confounders may influence treatment assignment and outcomes, and residual confounding cannot be excluded. The 33-month median follow-up captures early-to-intermediate recurrences but may underestimate late recurrence rates relevant for overall survival assessment, as longer follow-up identified continued divergence in survival curves beyond 3 years. The propensity score-matched analysis of EBRT efficacy is limited by small sample size (28 pairs) and wide confidence intervals (HR 0.42, 95% CI 0.20–0.88). The non-EBRT group comprised VBT alone (*n* = 16), chemotherapy (*n* = 16), and observation (*n* = 11), reflecting real-world deviation from NCCN guidelines. This heterogeneity limits causal inference, and findings should be considered hypothesis-generating pending prospective validation. Single-center surgical and pathological practices may limit external validity, and the exclusion rate for non-endometrioid histology and incomplete data may introduce selection bias. The risk stratification model was developed and validated in the same cohort, risking overfitting despite bootstrap optimism correction (1,000 iterations, optimism-corrected C-index 0.72). External validation in independent multi-center cohorts is essential before clinical implementation. Our study period partially predated widespread implementation of sentinel lymph node mapping with ultrastaging, potentially affecting nodal risk assessment accuracy, and future analyses should evaluate whether deep CSI retains prognostic significance in sentinel node-staged cohorts. With only 71 deep CSI patients, subgroup analyses were underpowered, and the wide confidence intervals for some interactions reflect statistical uncertainty requiring validation.

## 5 Conclusion

Deep cervical stromal invasion is a key factor for risk stratification in endometrial cancer with cervical stromal involvement, with biological plausibility rooted in anatomical lymphatic dissemination pathways and tumor-intrinsic aggressive features. Integration of CSI depth with histological grade, lymphovascular space invasion status, and molecular classification enables clinically actionable risk stratification, identifying a high-risk subgroup with greater than 45% three-year recurrence risk. Our findings support the routine incorporation of quantitative CSI depth assessment into pathological reporting and multidisciplinary treatment planning, with specific consideration of external beam radiotherapy for deep CSI patients regardless of other risk factors. These data contribute to the evolving evidence base supporting anatomic-molecular integrated risk models for personalized adjuvant therapy selection in endometrial cancer.

## Data Availability

The original contributions presented in the study are included in the article/Supplementary material, further inquiries can be directed to the corresponding author.
