# Supplementary material for: Deep cervical stromal invasion predicts poor prognosis in endometrioid endometrial cancer with cervical stromal involvement: a risk-stratification model for adjuvant therapy decision-making in endometrial carcinoma
Source: Front Med (Lausanne). 2026 Sep 16;13:1851729. doi: 10.3389/fmed.2026.1851729 (PMC13624127; doi:10.3389/fmed.2026.1851729)
Supplement: Supplementary file 1 [file Table_1.docx]

****Supplementary Table 1.** Comparison of Multivariable Cox Regression Results: Full Cohort (n=412) vs. Node-Negative Cohort (n=378)**

| **Variable** | **Full Cohort (n=412)** | **Node-Negative Cohort (n=378)** | | |
| --- | --- | --- | --- | --- |
|  | HR (95% CI) | P-value | HR (95% CI) | P-value |
| ****Deep CSI (≥50% vs. <50%)**** | ****2.45 (1.52–3.95)**** | ****<0.001**** | ****2.38 (1.45–3.91)**** | ****<0.001**** |
| Age (per 10-year increase) | 1.12 (0.86–1.46) | 0.401 | 1.10 (0.83–1.45) | 0.512 |
| Histological grade |  | 0.012 |  | 0.018 |
| G1 | Reference |  | Reference |  |
| G2 | 1.52 (0.84–2.76) | 0.168 | 1.48 (0.80–2.74) | 0.211 |
| G3 | 2.38 (1.28–4.42) | 0.006 | 2.25 (1.18–4.29) | 0.014 |
| Myometrial invasion (≥50% vs. <50%) | 1.78 (1.08–2.94) | 0.024 | 1.72 (1.02–2.90) | 0.042 |
| LVSI |  | 0.008 |  | 0.012 |
| None | Reference |  | Reference |  |
| Focal (<4 spaces) | 1.72 (0.91–3.25) | 0.095 | 1.68 (0.87–3.24) | 0.123 |
| Substantial (≥4 spaces) | 2.56 (1.38–4.75) | 0.003 | 2.48 (1.30–4.73) | 0.006 |
| Molecular subtype |  | 0.015 |  | 0.021 |
| POLE-mutated | 0.42 (0.11–1.62) | 0.208 | 0.40 (0.10–1.58) | 0.189 |
| MMR-deficient | 1.12 (0.61–2.06) | 0.712 | 1.08 (0.58–2.02) | 0.801 |
| NSMP | Reference |  | Reference |  |
| p53-aberrant | 2.18 (1.15–4.12) | 0.017 | 2.05 (1.06–3.97) | 0.033 |
| Adjuvant therapy |  | 0.008 |  | 0.012 |
| Observation | Reference |  | Reference |  |
| VBT alone | 0.82 (0.45–1.50) | 0.522 | 0.85 (0.46–1.57) | 0.601 |
| EBRT ± VBT | 0.48 (0.26–0.88) | 0.018 | 0.52 (0.28–0.97) | 0.039 |
| Chemotherapy/Chemoradiotherapy | 0.45 (0.23–0.88) | 0.021 | 0.48 (0.24–0.96) | 0.038 |

****Note:**** CSI = cervical stromal invasion; LVSI = lymphovascular space invasion; VBT = vaginal brachytherapy; EBRT = external beam radiotherapy; NSMP = no specific molecular profile; HR = hazard ratio; CI = confidence interval. Multivariable models adjusted for all variables listed. The proportional hazards assumption was verified using Schoenfeld residuals (full cohort: global test P=0.42; node-negative cohort: global test P=0.38).
